# Empagliflozin Alleviates Hepatic Steatosis and Oxidative Stress via the NRF1 Pathway in High-Fat Diet-Induced Mouse Model of Metabolic Dysfunction-Associated Steatotic Liver Disease

**DOI:** 10.3390/ijms26094054

**Published:** 2025-04-25

**Authors:** Yu Jung Heo, Jieun Park, Nami Lee, Sung-E Choi, Ja Young Jeon, Seung Jin Han, Dae Jung Kim, Kwan Woo Lee, Hae Jin Kim

**Affiliations:** 1 Institute of Medical Science, Ajou University School of Medicine, Suwon 16499, Republic of Korea; gotonature@ajou.ac.kr; 2 Department of Endocrinology and Metabolism, Ajou University School of Medicine, Suwon 16499, Republic of Korea; 500215@aumc.ac.kr (J.P.); chamsaram6@ajou.ac.kr (N.L.); se777@hanmail.net (S.-E.C.); twinstwins@ajou.ac.kr (J.Y.J.); hsj@ajou.ac.kr (S.J.H.); djkim@ajou.ac.kr (D.J.K.); lkw65@ajou.ac.kr (K.W.L.)

**Keywords:** empagliflozin, metabolic dysfunction-associated steatotic liver disease, Nrf1, hepatic steatosis, oxidative stress

## Abstract

Empagliflozin (EMPA)—a sodium-glucose cotransporter type 2 inhibitor—reduces endoplasmic reticulum (ER) stress, oxidative stress, and inflammation during metabolic dysfunction-associated steatotic liver disease (MASLD) progression. However, the direct effects of EMPA on hepatic lipid metabolism and oxidative stress are unclear. Through the current study, we seek to explore the effects of EMPA on oxidative stress and related mechanisms in MASLD. To this end, MASLD was induced in C57BL/6J mice using a high-fat diet (HFD); nuclear respiratory factor 1 (NRF1) was downregulated via viral transduction (AAV8-shNrf1). Glucose homeostasis and liver histology were assessed, and oxidative stress and inflammation were measured. HFD-fed mice-derived liver tissue samples exhibited more lipid droplets, higher triglyceride levels, and elevated oxidative and ER stress than chow diet (CD)-fed mice. EMPA attenuated HFD-induced liver oxidative and ER stress. Additionally, the HFD significantly decreased NRF1 and Sirtuin (SIRT)7 expression compared with CD, which was rescued by EMPA treatment. However, these results did not affect insulin resistance or lipid synthesis-related changes upon EMPA treatment in the *Nrf1*-knockdown mice. Furthermore, EMPA alleviated HFD-induced hepatic steatosis and oxidative stress; however, these effects were lost in *Nrf1*-knockdown mice. Collectively, the results of this study suggest that EMPA ameliorates MASLD by reducing steatosis and attenuating oxidative stress via NRF1.

## 1. Introduction

Metabolic dysfunction-associated steatotic liver disease (MASLD) causes a wide range of pathological conditions, from steatosis and metabolic dysfunction-associated steatohepatitis (MASH) to liver fibrosis and cirrhosis and is the most common cause of MASH-related hepatocellular carcinoma (HCC) worldwide [1]. MASLD is characterized by the combined impact of impaired glycolipid metabolism with low-grade systemic inflammation, leading to comorbidities such as type 2 diabetes mellitus (T2DM), obesity, arterial hypertension, and dyslipidemia [2,3]. MASLD progresses through two stages: an initial lipid accumulation and insulin resistance that is followed by oxidative stress and inflammation development [4]. The generation of reactive oxygen species (ROS) and the balance between pro- and anti-inflammatory pathways are key determinants of whether MASLD progresses to more severe stages, such as MASH, fibrosis, or cirrhosis [5,6]. A comprehensive understanding of these mechanisms is crucial for developing effective therapies and interventions for MASLD.

Sodium-glucose transport protein 2 inhibitors (SGLT2is), initially developed as antidiabetic drugs that increase urinary glucose excretion, were subsequently found to offer cardiovascular and renal protective effects [7,8]. Additionally, SGLT2is improve hepatic steatosis and decrease plasma liver enzyme levels in patients with MASLD [9]. Empagliflozin (EMPA), an SGLT2i, induces weight loss and is considered a promising agent for treating MASLD [10]. Additionally, EMPA inhibits the progression of MASH and exerts anti-steatotic, anti-inflammatory, and oxidative stress-reducing effects [11]. However, the mechanisms through which EMPA improves inflammation and oxidative stress remain unclear.

Nuclear respiratory factor 1 (NRF1) is a transcription factor with a wide range of functions that, in addition to its initial functions in erythroid cells, is essential in regulating cellular stress responses, inflammation, and cell differentiation [12]. Low expression of NRF1 in hepatocytes can lead to MASH and hepatomas, making it a critical player in liver health [13]. Meanwhile, mammalian sirtuin 7 (SIRT7) participates in promoting deacetylation and is primarily localized in the nucleus [14]. Although SIRT7 is widely expressed across various organs and tissues, its functions remain poorly characterized. Notably, the interaction between SIRT7 and NRF1 is associated with aging, mitochondrial function, and genome stability [15]. The involvement of NRF1 and SIRT7 in liver diseases, including steatohepatitis and liver cancer, further highlights their potential as therapeutic intervention targets [15,16,17]. Further research is required to elucidate the full function of NRF1 and the network of genes regulated by it. Indeed, investigating the relationship between NRF1 and SIRT7 is essential for understanding metabolic diseases and developing potential treatments for obesity and T2DM.

Accordingly, in the current study, we investigate the effect of EMPA on the liver of mice with high-fat diet (HFD)-induced MASLD and the related mechanisms. The results show that EMPA ameliorated MASLD by reducing hepatic steatosis and attenuating oxidative stress via NRF1.

## 2. Results

### 2.1. Empagliflozin Attenuates HFD-Induced Liver Oxidative Stress and Endoplasmic Reticulum Stress

**Figure 1:**
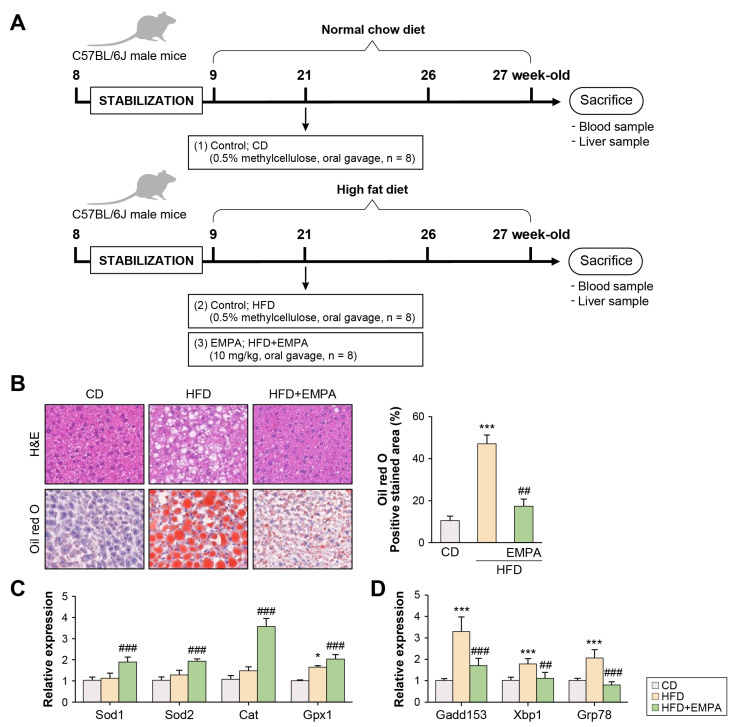
Effects of empagliflozin (EMPA) on oxidative stress and ER stress in the liver of high-fat diet-fed mice (**A**) EMPA was administered from week 21 to week 27. All mice were euthanized at the end of week 27. (**B**) Liver sections were assessed via H&E and Oil Red O (ORO) staining (red). Quantification of positive staining area was performed using ImageJ software (version 1.54g). (**C**) Relative expression of antioxidant enzyme mRNAs (*Sod1*, *Sod2*, *Cat*, and *Gpx1*) in the liver. (**D**) Expression of hepatic endoplasmic reticulum stress-related mRNAs (*Gadd153*, *Xbp1*, and *Grp78*) analyzed using quantitative real-time polymerase chain reaction. The results are presented as the mean ± standard deviation. * *p* < 0.05, and *** *p* < 0.001, control diet (CD) versus high-fat diet (HFD); ^##^ *p* < 0.01, and ^###^ *p* < 0.001, HFD diet versus HFD + EMPA.

To assess whether EMPA affects oxidative and/or endoplasmic reticulum (ER) stress, a mouse model was fed an HFD for 12 weeks and orally administered EMPA for 6 weeks (Figure 1A). EMPA-treated HFD mice exhibited decreased lipid accumulation compared with mice fed an HFD (Figure 1B). The expression of superoxide dismutase-1 (*Sod1*), *Sod2*, catalase (*Cat*), and glutathione peroxidase (*Gpx1*) mRNA was increased in EMPA-treated HFD mice compared with mice fed an HFD (Figure 1C). Meanwhile, EMPA treatment downregulated the expression of DNA damage-inducible gene-153 (*Gadd153*), X-box binding protein 1 (*Xbp1*), and chaperone glucose-regulated protein 78 (*Grp78*) in the liver compared with mice fed an HFD alone (Figure 1D). These results suggest that EMPA regulates oxidative and ER stress.

### 2.2. Empagliflozin Modulates NRF1 and SIRT7 Signaling Pathways in the Liver of HFD-Fed Mice

**Figure 2:**
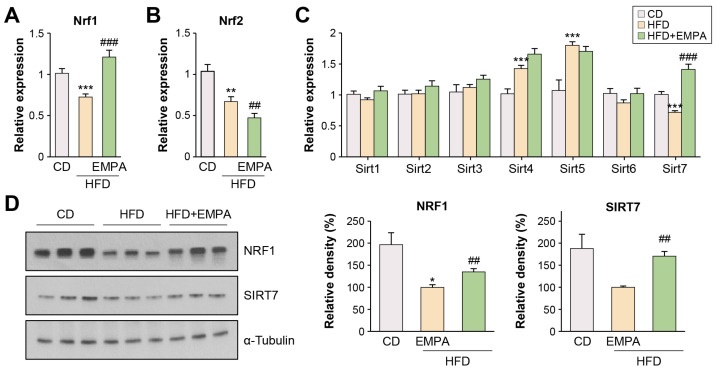
EMPA impacts the NRF1 and SIRT7 pathways in the liver of mice. (**A**) Relative expression of *Nrf1* in the liver. (**B**) Relative expression of *Nrf2* in the liver. (**C**) Quantitative analysis of Sirtuin mRNAs determined using quantitative polymerase chain reaction. (**D**) Representative hepatic expression of NRF1 and SIRT7 proteins determined using Western blot. The results are presented as the mean ± standard deviation. The maximum intensity of the EMPA-treated mice group was set to 100%, and the relative intensities of the experimental groups were calculated accordingly. * *p* < 0.05, ** *p* < 0.01, and *** *p* < 0.001, CD versus HFD diet; ^##^ *p* < 0.01, and ^###^ *p* < 0.001, HFD diet versus HFD + EMPA.

NRF1, NRF2, and SIRT7 participate in signaling pathways closely related to oxidative and ER stress [18,19]. Thus, EMPA-induced mRNA expression (*Nrf1*, *Nrf2*, and *Sirt*) was assessed in the livers of HFD-fed mice. Compared with mice fed the control diet (CD), those fed the HFD exhibited reduced *Nrf1* (Figure 2A), *Nrf2* (Figure 2B), and *Sirt7* (Figure 2C) mRNA expression and reduced NRF1 and SIRT7 protein levels (Figure 2D). EMPA-treated mice showed significantly increased *Nrf1* and *Sirt7* mRNA expression compared with the HFD group (Figure 2A,C). However, NRF1 and SIRT7 protein levels were lower in EMPA-treated mice than in the CD group but higher than in HFD mice that did not receive EMPA (Figure 2D). *Nrf2* mRNA expression was lower in the HFD group than in the CD group; EMPA treatment further reduced *Nrf2* expression (Figure 2B).

### 2.3. Empagliflozin Attenuates Hepatic Steatosis Through NRF1 in the Liver of HFD-Fed Mice

**Figure 3:**
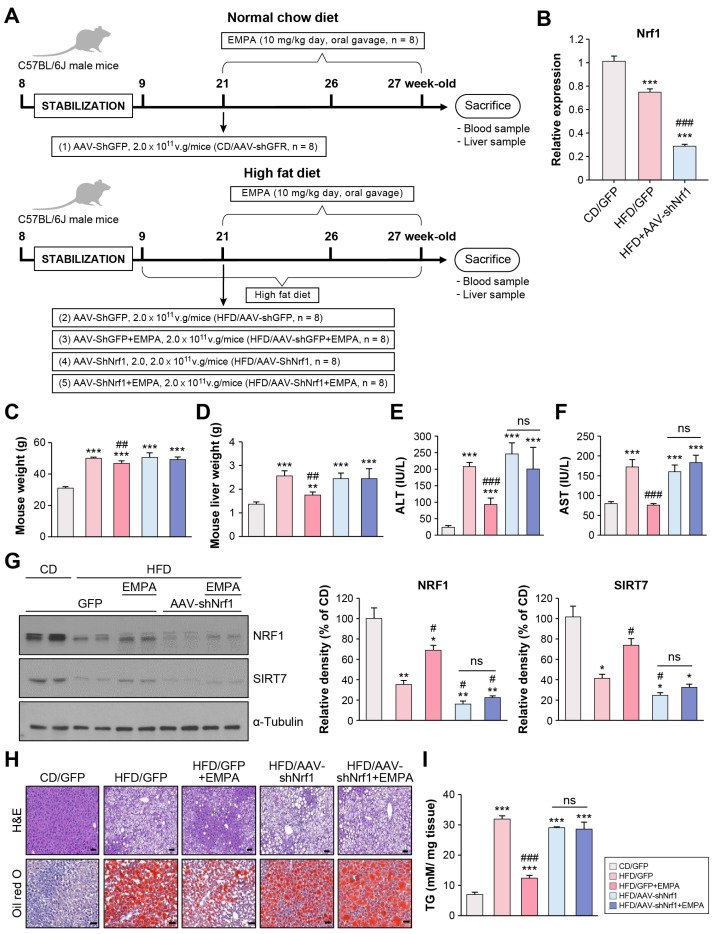
EMPA impacts hepatic steatosis through NRF1. (**A**) Male C57BL/6J mice were fed a control diet (CD) or high-fat diet (HFD) for 12 weeks. AAV-shRNA (2 × 10^11^ viral particles/mouse) was injected intravenously at 6 weeks and administrated EMPA. (**B**) mRNA expression of *Nrf1* in the liver of mice with AAV-GFP or AAV-shNrf1 transfection. Mouse weight (**C**) and liver weight (**D**). Plasma ALT (**E**) and AST (**F**) levels in mice. (**G**) Representative hepatic levels of NRF1 and SIRT7 proteins. Band intensities were measured with ImageJ software (version 1.54g). The maximum intensity of the CD group was set to 100%, and the relative intensities of the experimental groups were calculated accordingly. (**H**) Representative H&E and Oil Red O (ORO) staining (red) in mouse liver sections. Scale bar: 50 μm. (**I**) Hepatic triglyceride (TG) content. The results are presented as the mean ± standard deviation. * *p* < 0.05, ** *p* < 0.01, *** *p* < 0.001 vs. CD/GFP; ^#^ *p* < 0.05, ^##^ *p* < 0.01, and ^###^ *p* < 0.001 vs. HFD/GFP.

Sustained HFD intake induces substantial lipid accumulation in the liver [20]. To determine whether EMPA ameliorates liver injury via NRF1, shNrf1-expressing AAV (AAV-shNrf1) was injected into HFD-fed C57BL/6J mice (Figure 3A). Following AAV-shNrf1 injection, hepatic *Nrf1* mRNA expression was significantly reduced (Figure 3B). The bodyweight of all HFD groups (HFD/green fluorescent protein (GFP); HFD/GFP + EMPA; HFD-AAV shNrf1; HFD/AAVshNrf1 + EMPA) was significantly higher than the CD/GFP group. Among all HFD groups, the weight of the HFD/GFP + EMPA group was significantly lower than the HFD/GFP group (Figure 3C). A similar trend was observed for the liver weight (Figure 3D), alanine aminotransferase (ALT; Figure 3E), and aspartate aminotransferase (AST) levels (Figure 3F). Thus, EMPA had no effect in mice with NRF1 knockdown.

Given that EMPA reduced the expression of NRF1 and SIRT7, the effect of NRF1 knockdown on SIRT7 expression was investigated. NRF1 and SIRT7 protein levels were significantly lower in all HFD groups than in the CD/GFP group. NRF1 and SIRT7 protein levels in the HFD/GFP + EMPA group were significantly higher, while those in the HFD-AAV shNRF1 and HFD-AAV shNRF1 + EMPA groups were significantly lower than in the HFD/GFP group (Figure 3G). H&E and Oil Red O (ORO) staining revealed large lipid droplets in the liver tissues of HFD/GFP mice. EMPA treatment reduced hepatic lipid accumulation (Figure 3H). Additionally, all HFD-fed mice had significantly greater triglyceride (TG) levels than the CD/GFP group. The HFD/GFP + EMPA group had significantly lower TG levels than the HFD/GFP group. However, the TG levels in the HFD/AAVshNrf1 and HFD/AAVshNrf1 + EMPA groups did not differ significantly from the HFD/GFP group (Figure 3I). Meanwhile, no changes were observed in the liver weight, ALT, or AST levels between the CD/GFP and HFD/AAV-shNrf1 groups, with no changes in lipid droplet formation or TG levels. Of note, there was no significant difference between the HFD/AAVshNrf1 and HFD/AAVshNrf1 + EMPA groups in any of the abovementioned parameters, indicating that EMPA did not have any effect under *Nrf1* deficiency. These results suggest that EMPA regulates lipid steatosis via NRF1.

### 2.4. Empagliflozin Alleviates HFD-Induced Insulin Resistance and Lipid Synthesis via NRF1 in the Liver

**Figure 4:**
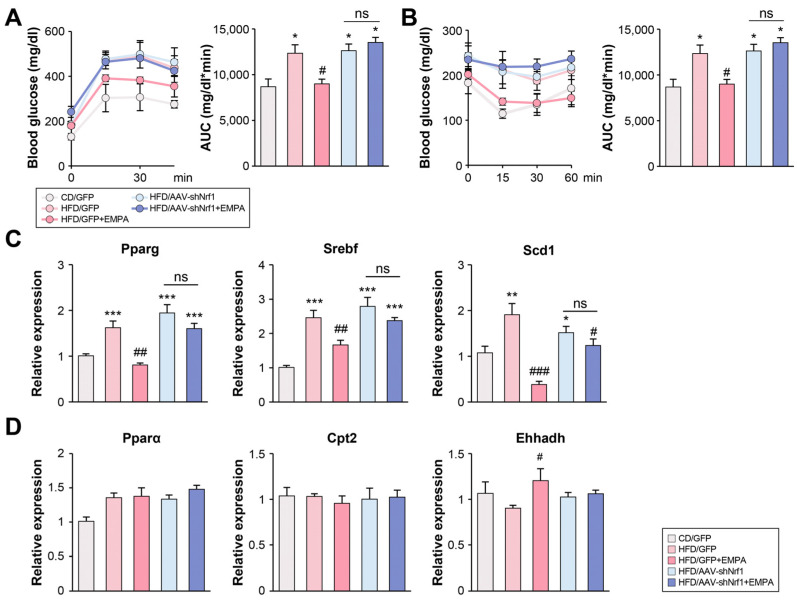
EMPA impacts insulin resistance and lipid synthesis through NRF1. (**A**) GTT and (**B**) ITT results after 6 h of fasting. Relative mRNA expression levels of key genes involved in (**C**) lipogenesis (*Pparg*, *Srebf*, *Scd1*) and (**D**) fatty acid oxidation (*Ppara*, *Cpt2*, *Ehhadh*) were measured in the liver. * *p* < 0.05, ** *p* < 0.01, *** *p* < 0.001 vs. CD/GFP; ^#^ *p* < 0.05, ^##^ *p* < 0.01, and ^###^ *p* < 0.001 vs. HFD/GFP. ns, not significant.

Insulin resistance is closely associated with lipid metabolism [3]. Compared with the CD/GFP group, glucose tolerance (GTT) (Figure 4A) and insulin tolerance (ITT) (Figure 4B) were significantly impaired in the HFD/GFP, HFD/AAVshNrf1, and HFD/AAVshNrf1 + EMPA groups. However, the HFD/GFP + EMPA group showed significantly alleviated glucose and insulin tolerance compared with the HFD/GFP group. Of note, there was no significant difference between the HFD/AAVshNrf1 and HFD/AAVshNrf1 + EMPA groups. The mRNA expression of genes involved in lipid synthesis, including peroxisome proliferator-activated receptor-gamma (*Pparg)*, sterol regulatory element-binding transcription factor *(Srebf)*, and stearyl CoA desaturase 1 (*Scd1)* were significantly higher in the HFD/GFP group than in the CD/GFP group. The expression in the HFD/GFP + EMPA group was significantly lower than in the HFD/GFP group, while there was no significant difference between the HFD/AAVshNrf1 and HFD/AAVshNrf1 + EMPA groups (Figure 4C). The mRNA expression of genes involved in fatty acid oxidation, such as *Ppara*, carnitine palmitoyltransferase 2 (*Cpt2)*, and enoyl-CoA hydratase and 3-hydroxyacyl CoA dehydrogenase (*Ehhadh)* did not differ significantly between any of the groups. Similarly, no change in the expression of fatty acid oxidation-related mRNA was detected between the CD/GFP and HFD/AAV-shNrf1 groups. The only exception to this was *Ehhadh*, which was upregulated in the HFD/GFP + EMPA group compared with the HFD/GFP group (Figure 4D). These results suggest that EMPA treatment of *Nrf1*-knockdown mice did not affect insulin resistance or lipid synthesis.

### 2.5. Empagliflozin Attenuates HFD-Induced Liver ER Stress and Oxidative Stress via NRF1

**Figure 5:**
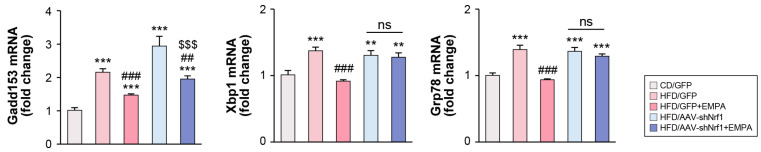
EMPA ameliorates ER stress through NRF1. Expression of hepatic endoplasmic reticulum (ER) stress-related genes (*Gadd153*, *Xbp1*, and *Gpr78*). The results are presented as the mean ± standard deviation. ** *p* < 0.01, and *** *p* < 0.001 vs. CD/GFP; ^##^ *p* < 0.01, and ^###^ *p* < 0.001 vs. HFD/GFP; ^$$$^ *p* < 0.001 vs. HFD/AAV-shNrf1. ns, not significant.

To evaluate the possible role of NRF1 in regulating ER stress induced by EMPA, *Nrf1*-knockdown mice were treated with or without EMPA. The expression of *Gadd153*, *Xbp1*, and *Grp78* was significantly higher in the HFD/GFP group than in the CD/GFP group. Meanwhile, their expression was downregulated in the HFD/GFP + EMPA group compared with the HFD/GFP group. Further, the expression of all genes in the HFD/AAVshNrf1 and HFD-AAVshNfr1 + EMPA group was significantly higher than in the CD/GFP group. However, no significant differences were observed between the HFD/AAVshNrf1 and HFD-AAVshNfr1 + EMPA groups, with the exception of *Gadd153*. Interestingly, *Gadd153* was upregulated in the HFD/AAV-shNrf1 group compared with the CD/GFP group (Figure 5).

**Figure 6:**
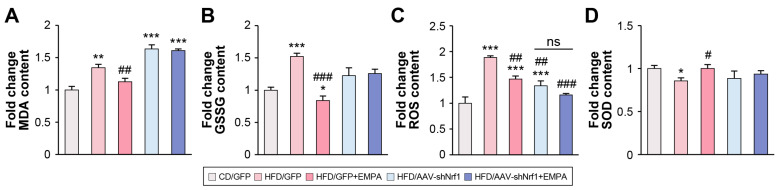
EMPA regulates oxidative stress through NRF1. (**A**) Analysis of the hepatic lipid peroxidation measured as MDA content in mice liver. (**B**) GSSG levels in liver homogenates. (**C**) ROS from liver tissues. (**D**) SOD levels in liver homogenates. * *p* < 0.05, ** *p* < 0.01, and *** *p* < 0.001 vs. CD/GFP; ^#^ *p* < 0.05, ^##^ *p* < 0.01, and ^###^ *p* < 0.001 vs. HFD/GFP. ns, not significant.

Liver oxidative stress was assessed to determine the effect of treatment on lipid peroxidation (malondialdehyde [MDA] content) and oxidized glutathione (GSSG) levels in the liver. The MDA content in the HFD/GFP group was significantly higher than in the CD/GFP group, while that in the HFD/GFP + EMPA group was significantly lower than in the HFD/GFP group. Further, the MDA content in the HFD/AAVshNrf1 and HFD/AAVshNrf1 + EMPA groups was significantly higher than in the CD/GFP group; however, no significant difference was detected between the HFD/AAVshNrf1 and HFD/AAVshNrf1 + EMPA groups (Figure 6A). The GSSG content in the HFD/GFP group was significantly higher than in the CD/GFP group, and that in the HFD/GFP + EMPA group was significantly lower than in the HFD/GFP and CD/GFP groups. The GSSG content in the HFD/AAVshNrf1 and HFD/AAVshNrf1 + EMPA groups did not differ significantly from the CD/GFP group. Similarly, there was no significant difference between the HFD/AAVshNrf1 and HFD/AAVshNrf1 + EMPA groups (Figure 6B).

The ROS levels in the HFD/GFP group were significantly higher than in the CD/GFP group, and that in the HFD/GFP + EMPA group was significantly lower than in the HFD/GFP group. The HFD/AAVshNrf1 and HFD/AAVshNrf1 + EMPA groups had significantly lower ROS levels than the HFD/GFP group. Meanwhile, no significant difference was detected between the HFD/AAVshNrf1 and HFD/AAVshNrf1 + EMPA groups (Figure 6C).

SOD is an important enzyme in the antioxidant system, protecting cells from oxidative damage [5]. The SOD levels in the HFD/GFP group were significantly lower than in the CD/GFP group. However, the HFD/GFP + EMPA group had a significantly higher SOD level than the HFD/GFP group. The HFD/AAVshNrf1 and HFD/AAVshNrf1 + EMPA groups had slightly lower SOD levels than the CD/GFP group, though this difference was not significant. Importantly, there was no difference between the HFD/AAVshNrf1 and HFD/AAVshNrf1 + EMPA groups (Figure 6D). These results suggest that EMPA regulates oxidative stress via NRF1.

## 3. Discussion

This study showed that EMPA treatment attenuated HFD-induced MASLD by activating NRF1 signaling to alleviate abnormal liver function and oxidative stress. These results demonstrate the critical role of NRF1 in EMPA-mediated changes in lipid metabolism and oxidative stress in a mouse model of MASLD.

The global prevalence of MASLD has recently increased sharply [1]. Its pathogenesis is complex and intricately linked to abnormal fat accumulation in the liver, i.e., hepatic steatosis, and systemic and hepatic insulin resistance [4]. In this study, HFD-fed mice exhibited serum and hepatic lipid dysfunction, increased body weight and fat mass, and abnormal liver function, with increased lipid droplets and significant steatosis, confirming that HFD administration can induce MASLD.

EMPA has proven effective in clinical trials for diabetes, cardiovascular disease, and MASLD [8]. EMPA improves liver dysfunction or severe liver pathology by reducing body weight, transaminase activity, fatty liver index, inflammatory responses, steatosis, and fibrosis [9,10,11]. The beneficial effects of EMPA may include a reduction in lipid synthesis and oxidative stress [21,22], though the mechanisms underlying these effects are not fully understood. Our results show that EMPA improved liver function and alleviated hepatic lipid accumulation in mice with HFD-induced MASLD. Moreover, lipid synthesis and oxidative stress were increased in mice fed an HFD and reduced after EMPA treatment. The dose of EMPA used in this study was established based on previously reported pharmacokinetic properties, efficacy, and safety data. Generally, the approved clinical dose of EMPA ranges from 10 to 25 mg/day, while in rodent models, doses are typically set within the range of 1–30 mg/kg [23]. Thus, 10 mg/kg was applied in the current study, with oral administration and an every-other-day regimen to ensure safety and efficacy.

Oxidative stress is a major pathological component of MASLD. Given that NRF1 is a stress-responsive gene involved in the antioxidant processes [12], in the current study, we investigated the NRF1-related mechanism through which EMPA protects against MASLD-associated oxidative stress and ER stress. Oxidative stress activates the transcription of various antioxidant genes via a cis-acting sequence known as the antioxidant response element (ARE). NRF1 and NRF2 are expressed in myriad tissues and cell types and bind to ARE as heterodimers with small musculoaponeurotic fibrosarcoma proteins [24]. The importance of NRF2 in oxidative stress responses is well established, and its dysfunction has been implicated in many diseases, including neurodegenerative disorders [25], cardiovascular diseases [26], and cancer [27]. Meanwhile, NRF1, although less extensively studied than NRF2, has also emerged as a key regulator in various physiological processes. Tissue-specific knockout studies have demonstrated that NRF1 is essential for maintaining liver function and contributes to the pathogenesis of steatohepatitis (fatty liver inflammation) and cancer. The involvement of NRF1 in cellular stress responses, inflammation, and tissue differentiation has been gaining recognition [28,29]. The current study revealed that EMPA reduced oxidative stress markers (MDA, GSSG, ROS); however, when *Nrf1* expression was inhibited, EMPA failed to reduce oxidative stress. Additionally, EMPA increased the expression of the antioxidant enzyme SOD, an effect inhibited by *Nrf1* knockdown. These findings suggest that NRF1 is critical in EMPA-induced oxidative stress regulation.

Furthermore, EMPA reduced the expression of ER stress-related markers GADD153, XBP1, and GRP78. While XBP1 and GRP78 expression was unaffected by *Nrf1* inhibition, GADD153 was downregulated following *Nrf1* inhibition and EMPA treatment. NRF1 may interact with members of the ATF4–CHOP (GADD153) pathway [30], influencing ER stress regulation. Therefore, inhibiting NRF1 may lead to reduced GADD153 expression. In contrast, XBP1 and GRP78 are primarily regulated via the inositol-requiring enzyme 1 (IRE1) signaling pathway and are more likely to be influenced by ATF6 and IRE1 rather than NRF1. Therefore, NRF1 inhibition is not expected to significantly affect the expression of XBP1 and GRP78. Accordingly, the observation that EMPA treatment following *Nrf1* inhibition selectively reduced GADD153 expression while not affecting XBP1 or GRP78 can be interpreted as a result of the distinct regulatory mechanisms.

Lu et al. reported that nobiletin supplementation effectively protects against alcohol-induced mitochondrial dysfunction, oxidative stress, and liver injury by modulating the hepatic NRF1–mitochondrial transcription factor A (TFAM) signaling pathway [31]. NRF1 is an important transcription factor primarily responsible for positively regulating genes related to mitochondrial biogenesis, including *Tfam* [32]. Silencing *Nrf1* results in a corresponding knockdown of *Tfam* expression and a decrease in mitochondrial DNA (mtDNA) content [33]. Moreover, alcohol-induced reduction in hepatic NRF1 levels correlates with ER stress-mediated TFAM reduction and mitochondrial dysfunction in mice [34]. Our results show that EMPA increased TFAM protein levels; however, TFAM expression was unchanged in HFD-fed mice with downregulated *Nrf1*.

NRF2 also exerts regulatory effects on the expression of multiple detoxification and antioxidant defense genes and is widely involved in protective mechanisms against various liver diseases [35,36]. Typically, a reduction in oxidative stress induced by EMPA would be expected to increase NRF2 expression; however, we found that, while NRF1 was activated, NRF2 signaling was downregulated. A recent report suggested that NRF2/heme oxygenase-1 (HO-1) activation exacerbates liver injury rather than exerting a protective effect against cholestatic liver injury (CLI). Inhibition of HO-1 or improved bilirubin transport alleviated liver injury in a CLI model. Meanwhile, *Nrf2* knockout conferred hepatoprotective effects in CLI mice and exacerbated liver injury in non-CLI mice. In CLI, oxidative stress activates NRF2/HO-1, causes bilirubin accumulation, and impairs mitochondrial function [37]. Our results show that *Nrf2* and HO-1 expression was reduced by EMPA. Hence, a decrease in NRF2 may, under certain conditions, exert beneficial effects. Although we investigated the effect of EMPA on oxidative stress and confirmed that it exerted its activity through NRF1, further studies are needed to elucidate the mechanism of EMPA via *Nrf2* silencing in vivo.

SIRT7 is a class III histone deacetylase that participates in RNA transcription, DNA damage response, lipid metabolism, bone formation, and immune regulation [38]. Shin et al. have reported that SIRT7 is induced by XBP1 upon ER stress and is recruited to the promoters of ribosomal proteins via Myc to repress ER stress-related gene expression and alleviate ER stress [39]. Mohrin et al. have reported that the regulatory pathway of the mitochondrial unfolded protein response (UPRmt) is mediated by the SIRT7 and NRF1 interaction, which is. closely associated with cellular energy metabolism and proliferation [15]. We found that EMPA upregulated *Nrf1* to alleviate liver injury while modulating downstream signaling, including that of SIRT7. In vivo, lipid metabolism and oxidative stress can be improved by EMPA. However, EMPA-mediated changes in lipid metabolism and oxidative stress were not altered by *Nrf1*-knockdown. In our study, experiments were not performed in *Nrf1* knock-out mice, and a clear study of the interaction between NRF1 and SIRT7 was not fully explored. Nevertheless, our results suggest that the effect of EMPA is dependent on *Nrf1* expression during MASLD metabolism.

Lipid metabolism includes fatty acid and TG metabolism and is involved in triglyceride accumulation, fatty acid oxidation, and de novo lipogenesis [40]. Herein, we found that EMPA decreased lipid synthesis, which is associated with decreased expression of *Ppara*, *Srebf*, and *Scd1*. Ma et al. have reported that EMPA regulates lipid oxidation by upregulating *Gpx4* and *Ppara* [41]. We confirmed that EMPA inhibited lipid synthesis, though lipid oxidation did not improve after EMPA treatment. Previous studies have demonstrated that hepatocyte-specific *Nrf1* knockout mice (Nrf1FF:AlbCre) exhibit a NASH-like phenotype even under a standard chow diet [28]. However, our results suggest that inhibiting *Nrf1* in HFD-fed mice did not significantly alter the NASH phenotype. This discrepancy may stem from the already altered metabolic state in HFD-fed mice, where compensatory mechanisms could mitigate the impact of *Nrf1* inhibition, or where pre-existing metabolic disturbances may overshadow its effects. Further research under diverse experimental conditions is necessary to clarify these findings.

Our results also show that EMPA ameliorated hepatic steatosis and decreased TG levels. Knocking down *Nrf1* exacerbated hepatic steatosis and increased TG levels. In mice treated with EMPA after downregulating *Nrf1*, there was no reduction in lipid accumulation or TG levels. Moreover, EMPA improved insulin resistance in mice with HFD-induced MASLD; however, this effect was not observed after *Nrf1* knockdown. Meanwhile, following EMPA treatment, *Nrf1* upregulation enhanced SIRT7 signaling pathway activation, protected against lipid deposition-related metabolic disorders, and inhibited lipogenesis and oxidative stress.

In summary, EMPA activates NRF1 and SIRT7, reduces lipid accumulation, improves insulin resistance, and suppresses oxidative stress in an HFD-fed mouse model of MASLD. However, these effects of EMPA are lost following *Nrf1* knockdown. Therefore, we suggest that EMPA ameliorates MASLD via NRF1, advancing our understanding of metabolic diseases.

## 4. Materials and Methods

### 4.1. Animal Study

Eight-week-old male C57BL/6J mice were obtained from Guangdong GemPharmatech (Guangzhou, China). All animals were housed under controlled temperature conditions (22 ± 2 °C) and a 12 h light/12 h dark cycle. After a one-week acclimatization period, they were provided ad libitum access to water and administered either a 60% fat-containing HFD or a 10% fat-containing standard chow diet (D12492 and D12450B, respectively, Research Diets, New Brunswick, NJ, USA).

In the first animal study, mice were fed an HFD for 18 weeks and orally administered EMPA (10 mg/kg) every other day for 6 weeks.

In the second animal study, the mice were divided into five groups: (1) AAV8-shGFP-injected, CD group (CD/AAV-shGFP; n = 8); (2) AAV8-shGFP-injected, HFD group (HFD/AAV-shGFP; n = 8); (3) AAV8-shGFP-injected + EMPA, HFD group (HFD/AAV-shGFP + EMPA; n = 8); (4) AAV8-shNrf1-injected, HFD group (HFD/AAV-shNrf1; n = 8); (5) AAV8-shNrf1-injected + EMPA, HFD group (HFD/AAV-shNrf1 + EMPA; n = 8). These mice were fed an HFD for 18 weeks. Six weeks prior to euthanasia, they were intravenously injected with AAV8-shGFP or AAV8-shNrf1 (2 × 10^11^ active viral particles in 200 μL saline per mouse), followed by oral administration of 10 mg/kg EMPA every other day.

All procedures were carried out following the Ajou Institutional Animal Care guidelines and ethical approval was granted by the Ajou Institutional Animal Care Committee (approval number: 2023-0083, approval date: 12 November 2023).

### 4.2. Construction of a shNrf1-Expressing AAV (AAV-shNrf1)

To knock down *Nrf1* in the liver, the plasmid vector pAAV[shRNA]-EGFP-U6 > mNrf1 [shRNA#1] (VB900146-2958tvu, VectorBuilder, Chicago, IL, USA) was constructed, and *Nrf1*-knockdown AAVs (AAV-shNrf1) were produced using the AAV8 packaging system (VectorBuilder, Chicago, IL, USA). The target sequence of shNrf1 was 5′-CTGCGCCACAGGAGGTTAATT-3′, located at nucleotides 388–435 of the mouse *Nrf1* gene (VB900146-2958tvu, VectorBuilder, Chicago, IL, USA).

### 4.3. Glucose and Insulin Tolerance Test

Mice were intraperitoneally injected with glucose (0.5 g/kg) or insulin (0.375 U/kg) after 6 h of fasting. Blood samples were collected from the tail vein before and 15, 30, 60, 90, and 120 min after administration. and blood glucose was measured using the Accu-Chek device (Korea Roche Diagnostics, Seoul, Republic of Korea).

### 4.4. Histological Analysis

Mouse liver samples were fixed in 10% formalin, routinely dehydrated, embedded in paraffin, sliced into 5 μm-thick sections, mounted on slides, and sequentially stained with an H&E solution (Abcam, Cambridge, UK). Frozen samples were prepared in 30% sucrose dissolved in phosphate-buffered saline (PBS). Liver tissue, frozen in the optimal cutting temperature compound, was cut into 5 µm-thick sections and stained with ORO solution (0.5% in 60% isopropanol solution) for 10 min. The tissue sections were rinsed thrice with water, and the nuclei were stained by immersing the tissue slides in a hematoxylin solution for 1 min. The red oil droplets were observed under a microscope using an Aperio ScanScope CS slide scanner (Leica, Wetzlar, Germany).

### 4.5. Measurement of TG Levels and Hepatic Biochemical Analysis

The mice were diet-restricted 6 h prior to euthanasia. Blood samples were collected from the heparinized solutions of the hearts, centrifuged at 1500× *g* and 4 °C for 15 min, and the plasma was stored at −80 °C.

Tissue TG level measurements were made using an EZ-Triglyceride Quantification Assay Kit (DOGEN, Seoul, Republic of Korea) according to the manufacturer’s instructions. Absorbance was measured at 570 nm using a microplate reader (Bio-Rad, Hercules, CA, USA). The blood ALT and AST level measurements were performed in a central laboratory (SCL Healthcare, Seoul, Republic of Korea).

### 4.6. RNA Isolation and Real-Time PCR

Total RNA was extracted using the RNAiso Plus reagent (TaKaRa Bio, Otsu, Japan). The isolated RNA was reverse transcribed to create cDNA using the PrimeScript^TM^ RT Master Mix (TaKaRa Bio, Otsu, Japan). Quantitative real-time PCR was performed using the primers listed in Table 1. Gene expression levels were normalized to those of the reference gene.

### 4.7. Western Blotting

Liver samples were electrophoresed on 4–12% acrylamide gels and transferred onto polyvinylidene difluoride membranes (Millipore, Billerica, MA, USA). The amount of protein was determined via Ponceau staining. The membranes were blocked with 5% (*w/v*) bovine serum albumin for 30 min and incubated overnight at 4 °C with the following primary antibodies: rabbit anti-NRF1 (#46743), rabbit anti-SIRT7 (#5360), and rabbit anti-α-tubulin (#2144), each obtained from Cell Signaling Technology, Inc. (Danvers, MA, USA). The membranes were incubated with the secondary antibody (horseradish peroxidase-conjugated anti-rabbit IgG) in 5% skim milk. Protein bands were visualized using enhanced chemiluminescence (Amersham Pharmacia Biotech, Piscataway, NJ, USA).

### 4.8. Biochemical Analysis

Liver samples stored at −80 °C were sectioned into 100 mg slices and placed in a 5% MPA solution for homogenization. The homogenate was centrifuged at 10,000× *g* for 10 min at 4 °C, and the supernatant was collected and stored at −80 °C. ROS levels were assessed by homogenizing 100 mg of liver tissue in 1 mL of PBS. The ROS/reactive nitrogen species concentrations in the supernatant were determined using a commercially available assay kit (Oxiselect^TM^ In Vitro ROS/RNS assay, CellBiolabs, San Diego, CA, USA) based on radical-mediated conversion of a fluorogenic substrate, according to the manufacturer’s instructions.

### 4.9. Statistical Analysis

The PRISM10 software (version 10.4.1, GraphPad Software, San Diego, CA, USA) was used for statistical analysis. Statistical differences were calculated using a two-way or one-way analysis of variance (ANOVA). The results are presented as mean ± standard deviation. *p*-values < 0.05 were considered significant.

## Figures and Tables

**Table 1:** Primer sequences used for quantitative real-time PCR.

Gene	Primer Forward	Primer Reverse
*Sod1*	GCTCCCAGCATTTCCAGTCT	TAACTGAAGGCCAGCATGGG
*Sod2*	GCTTGATAGCCTCCAGCAAC	CCGAGGAGAAGTACCACGAG
*Cat*	GAGTGTCCGGGTAGGCAAAA	TTCGTCCCGAGTCTCTCCAT
*GPx1*	TGTCGATGGTACGAAAGCGG	CAGTCCACCGTGTATGCCTT
*Gadd153*	CAGGGTCAAGAGTAGTGAAGGT	CTGGAAGCCTGGTATGAGGAT
*Xbp1*	ACAGGGTCCAACTTGTCCAG	TCCGCAGCACTCAGACTATG
*Grp78*	CTTCATAGTCCTGCCCATTG	CGAAGGGATCATCTGCTATTAC
*Nrf1*	AGGTGGTGACCTTGGAACAG	GGCTTTTTGGGACAGTGAAA
*Nrf2*	TTCGTTAAAGGGGAGGGACT	GGAAAGGCACAGAGAGCATC
*Sirt1*	TCCACGGTGCTGAGGTATAC	GCCACTGTCACTGTTACTGC
*Sirt2*	AGCCAACCATCTGCCACTAC	CACTCGTTCCAGCGTGTCTA
*Sirt3*	TTTCTTTCACAACCCCAAGC	TGGAGGAGCCTCAGGAAGTA
*Sirt4*	CATCCAGCACATTGATTTCG	GTTGGGTTGGTGAGAGGAGA
*Sirt5*	CCACCGACAGATTCAGGTTT	GGGCGGTTAAGAAGTCCTTT
*Sirt6*	CCCAAGTTTGACACCACCTT	CTGACCAGGAAGCTGAGGAA
*Sirt7*	GACTGAGCGTACTGCCCTTC	ACAATGGTATCCCGAAGCTG
*Pparg*	TTCAGAAGTGCCTTGCTGTG	CCAACAGCTTCTCCTTCTCG
*Ppara*	CGAGGTGAAAGATTCGGAAA	GGCCTTGACCTTGTTCATGT
*Srebf*	GCTGTTGGCATCCTGCTATC	AGCTGGAAGTGACGGTGGT
*Cpt2*	GGATTTTGAGAACGGCATTGG	TCATCACGACTGGGTTTGGGT
*Ehhadh*	CTGGCTATGATCCGCCTCTG	CTGCGGGGTTCTATGGGTTT
*Scd1*	CACACGCCGACCCTCACAAT	TTTGACAGCCGGGTGTTTGC

*Sod*: superoxide dismutase; *Cat*: catalase; *GPx1*: glutathione peroxidase; *Gadd153*, growth arrest- and DNA damage-inducible gene 153; *Xbp1*: X-box binding protein 1; *Gpr78*: G protein-coupled receptor 78; *Nrf*: nuclear respiratory factor; *Sirt*: Sirtuin; *Ppar*: peroxisome proliferator-activated receptor; *Srebf*: sterol regulatory element-binding transcription factor 1; *Cpt2*: carnitine palmitoyltransferase II, *Ehhadh*: enoyl-CoA hydratase and 3-hydroxyacyl CoA dehydrogenase; *Scd1*: stearoyl-CoA desaturase-1.

## Data Availability

The original contributions presented in this study are included in the article. Further inquiries can be directed at the corresponding author.

## Abbreviations

The following abbreviations are used in this manuscript:

ALT	Alanine aminotransferase
ANOVA	Analysis of variance
AST	Aspartate aminotransferase
ARE	Antioxidant response element
CAT	Catalase
CD	Control diet
CLI	Cholestatic liver injury
CPT2	Carnitine palmitoyltransferase 2
EHHADH	Enoyl-CoA hydratase and 3-hydroxyacyl CoA dehydrogenase
EMPA	Empagliflozin
ER	Endoplasmic reticulum
GADD153	DNA damage-inducible gene-153
GFP	Green fluorescent protein
GPX-1	Glutathione peroxidase-1
GRP	Glucose-regulated protein
GSSG	Oxidized glutathione
HCC	Hepatocellular carcinoma
H&E	Hematoxylin and eosin
HFD	High-fat diet
HO-1	Heme oxygenase-1
IRE1	Inositol-requiring enzyme 1
MASH	Metabolic dysfunction-associated steatohepatitis
MASLD	Metabolic dysfunction-associated steatotic liver disease
MDA	Malondialdehyde
NRF-1	Nuclear respiratory factor 1
ORO	Oil Red O
PPAR	Peroxisome proliferator-activated receptor
ROS	Reactive oxygen species
SCD1	Stearyl CoA desaturase 1
SGLT2i	Sodium–glucose transport protein 2 inhibitor
SIRT	Sirtuin
SOD	Superoxide dismutase
SREBF	Sterol regulatory element-binding transcription factor
XBP-1	X-box binding protein 1
T2DM	Type 2 diabetes mellitus
TG	Triglyceride
TFAM	Mitochondrial transcription factor A
UPR	Unfolded protein response

## References

[1] Foglia B., Sutti S., Cannito S., Rosso C., Maggiora M., Casalino A., Bocca C., Novo E., Protopapa F., Ramavath N.N. (2024). Histidine-rich glycoprotein in metabolic dysfunction-associated steatohepatitis-related disease progression and liver carcinogenesis. Front. Immunol..

[2] Bray G.A., Heisel W.E., Afshin A., Jensen M.D., Dietz W.H., Long M., Kushner R.F., Daniels S.R., Wadden T.A., Tsai A.G. (2018). The science of obesity management: An endocrine society scientific statement. Endocr. Rev..

[3] Jung U.J., Choi M.S. (2014). Obesity and its metabolic complications: The role of adipokines and the relationship between obesity, inflammation, insulin resistance, dyslipidemia and nonalcoholic fatty liver disease. Int. J. Mol. Sci..

[4] Day C.P., James O.F. (1998). Steatohepatitis: A tale of two “hits”?. Gastroenterology.

[5] Arroyave-Ospina J.C., Wu Z., Geng Y., Moshage H. (2021). Role of oxidative stress in the pathogenesis of non-alcoholic fatty liver disease: Implications for prevention and therapy. Antioxidants.

[6] Rolo A.P., Teodoro J.S., Palmeira C.M. (2012). Role of oxidative stress in the pathogenesis of nonalcoholic steatohepatitis. Free Radic. Biol. Med..

[7] Zinman B., Wanner C., Lachin J.M., Fitchett D., Bluhmki E., Hantel S., Mattheus M., Devins T., Johansen O.E., Woerle H.J. (2015). Empagliflozin, cardiovascular outcomes, and mortality in type 2 diabetes. N. Engl. J. Med..

[8] McGuire D.K., Shih W.J., Cosentino F., Charbonnel B., Cherney D.Z.I., Dagogo-Jack S., Pratley R., Greenberg M., Wang S., Huyck S. (2021). Association of SGLT2 inhibitors with cardiovascular and kidney outcomes in patients with type 2 diabetes: A meta-analysis. JAMA Cardiol..

[9] Gastaldelli A., Cusi K. (2019). From NASH to diabetes and from diabetes to NASH: Mechanisms and treatment options. JHEP Rep..

[10] Kramer C.K., Zinman B. (2019). Sodium–glucose cotransporter–2 (SGLT-2) inhibitors and the treatment of type 2 diabetes. Annu. Rev. Med..

[11] Hsiang J.C., Wong V.W. (2020). SGLT2 inhibitors in liver patients. Clin. Gastroenterol. Hepatol..

[12] Biswas M., Chan J.Y. (2010). Role of Nrf1 in antioxidant response element-mediated gene expression and beyond. Toxicol. Appl. Pharmacol..

[13] Tsujita T., Peirce V., Baird L., Matsuyama Y., Takaku M., Walsh S.V., Griffin J.L., Uruno A., Yamamoto M., Hayes J.D. (2014). Transcription factor Nrf1 negatively regulates the cystine/glutamate transporter and lipid-metabolizing enzymes. Mol. Cell. Biol..

[14] Wu Q.-J., Zhang T.-N., Chen H.-H., Yu X.-F., Lv J.-L., Liu Y.-Y., Liu Y.-S., Zheng G., Zhao J.-Q., Wei Y.-F. (2022). The sirtuin family in health and disease. Signal Transduct. Target. Ther..

[15] Mohrin M., Shin J., Liu Y., Brown K., Luo H., Xi Y., Haynes C.M., Chen D. (2015). Stem cell aging. A mitochondrial UPR-mediated metabolic checkpoint regulates hematopoietic stem cell aging. Science.

[16] Akl M.G., Li L., Widenmaier S.B. (2024). Protective effects of hepatocyte stress defenders, Nrf1 and Nrf2, against MASLD progression. Int. J. Mol. Sci..

[17] Zeng C., Chen M. (2022). Progress in nonalcoholic fatty liver disease: SIRT family regulates mitochondrial biogenesis. Biomolecules.

[18] Digaleh H., Kiaei M., Khodagholi F. (2013). Nrf2 and Nrf1 signaling and ER stress crosstalk: Implication for proteasomal degradation and autophagy. Cell. Mol. Life Sci..

[19] Wu D., Li Y., Zhu K.S., Wang H., Zhu W.G. (2018). Advances in cellular characterization of the sirtuin isoform, SIRT7. Front. Endocrinol..

[20] Tsuru H., Osaka M., Hiraoka Y., Yoshida M. (2020). HFD-induced hepatic lipid accumulation and inflammation are decreased in Factor D deficient mouse. Sci. Rep..

[21] Hüttl M., Markova I., Miklankova D., Zapletalova I., Poruba M., Haluzik M., Vaněčkova I., Malinska H. (2021). In a prediabetic model, empagliflozin improves hepatic lipid metabolism independently of obesity and before onset of hyperglycemia. Int. J. Mol. Sci..

[22] Mone P., Varzideh F., Jankauskas S.S., Pansini A., Lombardi A., Frullone S., Santulli G. (2022). SGLT2 inhibition via empagliflozin improves endothelial function and reduces mitochondrial oxidative stress: Insights from frail hypertensive and diabetic patients. Hypertension.

[23] Nasiri-Ansari N., Nikolopoulou C., Papoutsi K., Kyrou I., Mantzoros C.S., Kyriakopoulos G., Chatzigeorgiou A., Kalotychou V., Randeva M.S., Chatha K. (2021). Empagliflozin Attenuates Non-Alcoholic Fatty Liver Disease (NAFLD) in High Fat Diet Fed ApoE^(-/-)^ Mice by Activating Autophagy and Reducing ER Stress and Apoptosis. Int. J. Mol. Sci..

[24] Katsuoka F., Motohashi H., Ishii T., Aburatani H., Engel J.D., Yamamoto M. (2005). Genetic evidence that small maf proteins are essential for the activation of antioxidant response element-dependent genes. Mol. Cell. Biol..

[25] Dinkova-Kostova A.T., Kostov R.V., Kazantsev A.G. (2018). The role of Nrf2 signaling in counteracting neurodegenerative diseases. FEBS J..

[26] Chen Q.M., Maltagliati A.J. (2018). Nrf2 at the heart of oxidative stress and cardiac protection. Physiol. Genom..

[27] Zimta A.-A., Cenariu D., Irimie A., Magdo L., Nabavi S.M., Atanasov A.G., Berindan-Neagoe I. (2019). The role of Nrf2 activity in cancer development and progression. Cancers.

[28] Xu Z., Chen L., Leung L., Yen T.S., Lee C., Chan J.Y. (2005). Liver-specific inactivation of the Nrf1 gene in adult mouse leads to nonalcoholic steatohepatitis and hepatic neoplasia. Proc. Natl. Acad. Sci. USA.

[29] Hu S., Feng J., Wang M., Wufuer R., Liu K., Zhang Z., Zhang Y. (2022). Nrf1 is an indispensable redox-determining factor for mitochondrial homeostasis by integrating multi-hierarchical regulatory networks. Redox Biol..

[30] Galehdar Z., Swan P., Fuerth B., Callaghan S.M., Park D.S., Cregan S.P. (2010). Neuronal apoptosis induced by endoplasmic reticulum stress is regulated by ATF4-CHOP-mediated induction of the Bcl-2 homology 3-only member PUMA. J. Neurosci..

[31] Lu D., Huang A., Tong X., Zhang X., Li S., Yu X. (2024). Nobiletin protects against alcohol-induced mitochondrial dysfunction and liver injury by regulating the hepatic NRF1-TFAM signaling pathway. Redox Rep..

[32] Gleyzer N., Vercauteren K., Scarpulla R.C. (2005). Control of mitochondrial transcription specificity factors (TFB1M and TFB2M) by nuclear respiratory factors (NRF-1 and NRF-2) and PGC-1 family coactivators. Mol. Cell. Biol..

[33] Piantadosi C.A., Suliman H.B. (2006). Mitochondrial transcription factor A induction by redox activation of nuclear respiratory factor 1. J. Biol. Chem..

[34] Hao L., Zhong W., Dong H., Guo W., Sun X., Zhang W., Yue R., Li T., Griffiths A., Ahmadi A.R. (2021). ATF4 activation promotes hepatic mitochondrial dysfunction by repressing NRF1-TFAM signalling in alcoholic steatohepatitis. Gut.

[35] Gao Z., Yi W., Tang J., Sun Y., Huang J., Lan T., Dai X., Xu S., Jin Z.-G., Wu X. (2022). Urolithin A protects against acetaminophen-induced liver injury in mice via sustained activation of Nrf2. Int. J. Biol. Sci..

[36] Rodríguez M.J., Sabaj M., Tolosa G., Vielma F.H., Zúñiga M.J., González D.R., Zúñiga-Hernández J. (2021). Maresin-1 prevents liver fibrosis by targeting Nrf2 and NF-κB, reducing oxidative stress and inflammation. Cells.

[37] Wang Y., Fu X., Zeng L., Hu Y., Gao R., Xian S., Liao S., Huang J., Yang Y., Liu J. (2024). Activation of Nrf2/HO-1 signaling pathway exacerbates cholestatic liver injury. Commun. Biol..

[38] Gu Y., Ding C., Yu T., Liu B., Tang W., Wang Z., Tang X., Liang G., Peng J., Zhang X. (2024). SIRT7 promotes Hippo/YAP activation and cancer cell proliferation in hepatocellular carcinoma via suppressing MST1. Cancer Sci..

[39] Shin J., He M., Liu Y., Paredes S., Villanova L., Brown K., Qiu X., Nabavi N., Mohrin M., Wojnoonski K. (2013). SIRT7 represses Myc activity to suppress ER stress and prevent fatty liver disease. Cell Rep..

[40] Carli F., Della Pepa G., Sabatini S., Puig A.V., Gastaldelli A. (2024). Lipid metabolism in MASLD and MASH: From mechanism to the clinic. JHEP Rep..

[41] Ma Y., Zhang G., Kuang Z., Xu Q., Ye T., Li X., Qu N., Han F., Kan C., Sun X. (2022). Empagliflozin activates Sestrin2-mediated AMPK/mTOR pathway and ameliorates lipid accumulation in obesity-related nonalcoholic fatty liver disease. Front. Pharmacol..

